# *Wolbachia *transmission dynamics in *Formica *wood ants

**DOI:** 10.1186/1471-2148-8-55

**Published:** 2008-02-21

**Authors:** Lumi Viljakainen, Max Reuter, Pekka Pamilo

**Affiliations:** 1 Department of Biology, PO Box 3000, 90014 University of Oulu, Finland; 2 The Galton Laboratory, Department of Biology, University College London, 4 Stephenson Way, London NW1 2HE, UK

## Abstract

**Background:**

The role of *Wolbachia *endosymbionts in shaping the mitochondrial diversity of their arthropod host depends on the effects they have on host reproduction and on the mode of transmission of the bacteria. We have compared the sequence diversity of *wsp *(*Wolbachia *surface protein gene) and the host mtDNA in a group of *Formica *ant species that have diverged approximately 0.5 million years ago (MYA). The aim was to study the relationship of *Wolbachia *and its ant hosts in terms of vertical and horizontal transmission of the bacteria.

**Results:**

All studied ant species were doubly infected with two *Wolbachia *strains (wFex1 and wFex4) all over their geographical distribution area in Eurasia. The most common haplotypes of these strains were identical with strains previously described from a more distantly related *Formica *ant, with an estimated divergence time of 3.5 – 4 MYA. Some strain haplotypes were associated to the same or closely related mtDNA haplotypes as expected under vertical transmission. However, in several cases the *wsp *haplotypes coexisted with distant mtDNA haplotypes, a pattern which is more compatible with horizontal transmission of the bacteria.

**Conclusion:**

Two lines of evidence suggest that the sharing of *Wolbachia *strains by all *F. rufa *species is rather due to horizontal than vertical transmission. First, the fact that endosymbiont strains identical to those of *F. rufa *ants have been found in another species that diverged 3.5–4 MYA strongly suggests that horizontal transfer can and does occur between *Formica *ants. Second, the frequent sharing of identical *Wolbachia *strains by distant mitochondrial lineages within the *F. rufa *group further shows that horizontal transmission has occurred repeatedly. Nevertheless, our dataset also provides some evidence for longer-term persistence of infection, indicating that *Wolbachia *infection within this host clade has been shaped by both horizontal and vertical transmission of symbionts. The fact that all the ants were infected irrespective of the family structure of their societies gives no support to the proposed hypotheses that the spreading of *Wolbachia *in ants might be associated to the types of their societies.

## Background

*Wolbachia*, the common endosymbiotic bacteria of arthropods and filarial nematodes, may have an influence on the genetic variation of its host population. Maternally transmitted *Wolbachia *causes, among others, cytoplasmic incompatibility (CI), which prevents reproduction between an uninfected female and an infected male, or between a female and a male harboring different *Wolbachia *strains [1,2]. As CI *Wolbachia *spreads in the population, the associated, also maternally inherited mitochondria are transmitted along with it. Theory predicts that the mitochondrial haplotype associated with successfully spreading *Wolbachia *will increase in frequency, causing a reduction in the overall mitochondrial variation [3], and finally leading to the elimination of haplotypes associated with uninfected females [4].

The effect of *Wolbachia *on host genetic variation depends on its transmission dynamics, i.e. whether it is strictly vertically transmitted from mother to offspring, or whether horizontal transmission also occurs between individuals or even between species. The reduction in mitochondrial variation caused by a selective sweep of *Wolbachia *occurs only if *Wolbachia *spread solely vertically [4]. Horizontal transmission of *Wolbachia *will weaken the association between *Wolbachia *and the mitochondrial haplotype, and the effects on host mitochondrial DNA variation are presumed to be negligible.

In addition to CI, *Wolbachia *can promote its own spread by manipulating host reproduction by inducing parthenogenesis, killing males, feminizing genetic males and have even shown to be obligate mutualists [5-7]. Hymenoptera have haplodiploid sex determination where haploid eggs develop to males and diploid eggs to females. In addition, in some Hymenoptera only a single locus is involved in the sex determination [8,9]. These systems rule out half of the *Wolbachia *manipulation types. Induction of parthenogenesis by automixis would lead to diploid offspring that are homozygous at the sex-determining locus and would develop into males instead of females, and diploid males are known to be inviable or sterile. Feminization is also ruled out as that would result in haploid females that are not able to reproduce. This leaves CI, male killing and host dependence as the modes of *Wolbachia*'s reproductive manipulation in hymenopteran social insects. CI has been reported to be the most common effect of *Wolbachia *on arthropod hosts [10], and that might be true also in ants [11-14].

The putative effects of *Wolbachia *on reproduction of hymenopteran social insects have raised great interest, as sex ratio manipulation is a central theme in social insect biology, predicted by the kin selection theory as part of the queen-worker conflicts [15]. Furthermore, it has been suggested that the spread and prevalence of *Wolbachia *infections could be associated to the social type of insect colonies [16,17] and to the invasion histories [18,19].

The aim of the present study was to shed light on the relationship between *Wolbachia *and the *Formica rufa *group wood ants by focusing on the transmission modes of the bacteria. *Wolbachia *infection status in the ants was defined by using a polymerase chain reaction (PCR) assay and the transmission dynamics investigated by comparing *Wolbachia *strains with associated mtDNA haplotypes. For this purpose we used the mitochondrial phylogeny of *F. rufa *group ants [20] based on samples from a large geographical scale covering most of the Eurasian distribution of the wood ants, and we screened *Wolbachia *in ants from the same nests used in the mtDNA study.

We found that all the *F. rufa *group ant species are infected with two common strains of *Wolbachia*. Two lines of evidence indicate that the generality of this double-infection is likely to be the result of horizontal transmission of *Wolbachia *between host species rather than the maintenance of an ancestral infection. Firstly, the same *Wolbachia *strains and haplotypes have been found in the more distantly related species *Formica exsecta *with a divergence time of 3.5 – 4 million years ago [21]. Secondly, rare *Wolbachia *haplotypes are shared by *Formica *wood ants with distant mtDNA haplotypes. However, the data shows some evidence for long-term persistence of *Wolbachia *infection in some lineages, suggesting that both horizontal and vertical transmission of *Wolbachia *have been important in the infection history of *Formica *ants. As all the ants were infected irrespective of their social organization, our data gives no support to the hypotheses that the spreading of *Wolbachia *in ants might be associated to the types of their societies.

## Results

### Infection status

All 32 wood ant samples of the six *F. rufa *group species contained *Wolbachia *(Table 1). Most of the ants carried a double infection of two group A *Wolbachia *strains, wFex1 and wFex4, which have both been previously identified in Swiss populations of the ant *F. exsecta *[22]. In addition, *F. rufa *contained group A strain wFex2 (also previously described from Swiss *F. exsecta *[22]) and a new strain named wFrufa [GenBank:EF554317]. All strains were clearly distinct in sequence, as the amount of nucleotide differences in the 546–567 bp long sequences ranged from 10% between wFex1 and wFex2 to 21% between wFex1 and wFex4.

**Table 1:** Study species, their locations and mtDNA haplotypes, and the number of different *Wolbachia *strains.

Species	Location	Sample	mtDNA haplotype^a^	wFex1^b^	wFex4^b^	Other strains^b^	Total
*F. aquilonia*	Sweden	S-113	P	6	14		20
*F. aquilonia*	Sweden	S-67-3	P	5	15		20
*F. aquilonia*	Russia, Novosibirsk	N-28-2	P	3	15		18
*F. aquilonia*	Russia, Ural	U-84-2	P	6	17	1^c^	24
*F. aquilonia*	Russia, Baikal	E-8	P	29	26		55
*F. aquilonia*	Russia, Baikal	E-5-2	B	26	33		59
*F. aquilonia*	Scotland	GB-5	P	-	20		20
*F. lugubris*	Scotland	GB-P20	N	6	14		20
*F. lugubris*	France	P-21	J	4	16		20
*F. lugubris*	Switzerland	CE-4	L	8	12		20
*F. lugubris*	Russia, Baikal	E-4	A	9	1		10
*F. lugubris*	Russia, Baikal	E-4-2	A	7	3		10
*F. lugubris*	Russia, Baikal	E-1	C	8	12		20
*F. lugubris*	Russia, Moscow	WE-2	E	10	10		20
*F. lugubris*	Russia, Moscow	WE2-2	E	5	5		10
*F. lugubris*	France	P-5	H	-	20		20
*F. lugubris*	France	P-5-2	H	7	13		20
*F. lugubris*	England	0-23-1	M	10	10		20
*F. paralugubris*	Switzerland	CE-1	O	6	4		10
*F. paralugubris*	Switzerland	CE-3	O	9	1		10
*F. polyctena*	Russia, Ural	U-23-4	O	8	2		10
*F. polyctena*	Russia, Ural	U-23-5	O	6	4		10
*F. polyctena*	Russia, Ural	U-15	Y	9	7		16
*F. polyctena*	Sweden	S-30	W	5	13		18
*F. pratensis*	Russia, Novosibirsk	N-17	T	8	12		20
*F. pratensis*	Russia, Baikal	E-10	V	7	3		10
*F. pratensis*	Russia, Baikal	E-10-2	V	3	7		10
*F. pratensis*	Finland	S-58	U	7	13		20
*F. pratensis*	France	P-19	I	7	5		12
*F. pratensis*	France	P-19-2	I	4	3		7
*F. rufa*	Russia, Novosibirsk	N-16	AD	4	-	7^d^	11
*F. rufa*	Russia, Ural	U-18	AA	7	8	1^d^,1^e^	17

				239	338	10	587

^a ^From [20].

^b ^the number of clones sequenced from each strain

^c ^wFex3, ^d ^wFex2, ^e ^wFrufa

The overall proportions of the two common *Wolbachia *strains wFex1 and wFex4 in the ant species sampled were 40% and 57%, respectively. Within species frequencies varied from 26% to 75% in wFex1, and from 25% to 70% in wFex4. The heterogeneity of the proportions of these two strains among the host species was close to significant (Kruskal-Wallis test, H = 10.45, df = 5, 0.05 < P < 0.10). Strain wFex1 was more common in *F. paralugubris *(75%), *F. polyctena *(55%) and *F. rufa *(39%), whereas wFex4 was the most frequent in *F. aquilonia *(70%), *F. lugubris *(57%) and *F. pratensis *(52%). Two samples, GB-5 (*F. aquilonia*) and P-5 (*F. lugubris*), contained only wFex4.

### Nucleotide variation within strains

Both wFex1 and wFex4 had one common haplotype found in most samples. Notably, in both cases the common haplotype was identical to that described from *F. exsecta *[22]. In addition to these common sequences, some rare *wsp *haplotypes were detected for both wFex1 and wFex4 in 25 samples (78% of the specimens), with 1–3 point mutations per haplotype in wFex1 and 1–4 in wFex4. As it was impossible to determine which mutations were errors made by the PCR polymerase enzyme, none of the singletons in *wsp *sequences were taken into account in further analysis. However, when the same mutation within a *Wolbachia *strain was found in at least two distinct ant samples, it was regarded as a true mutation.

**Figure 1:**
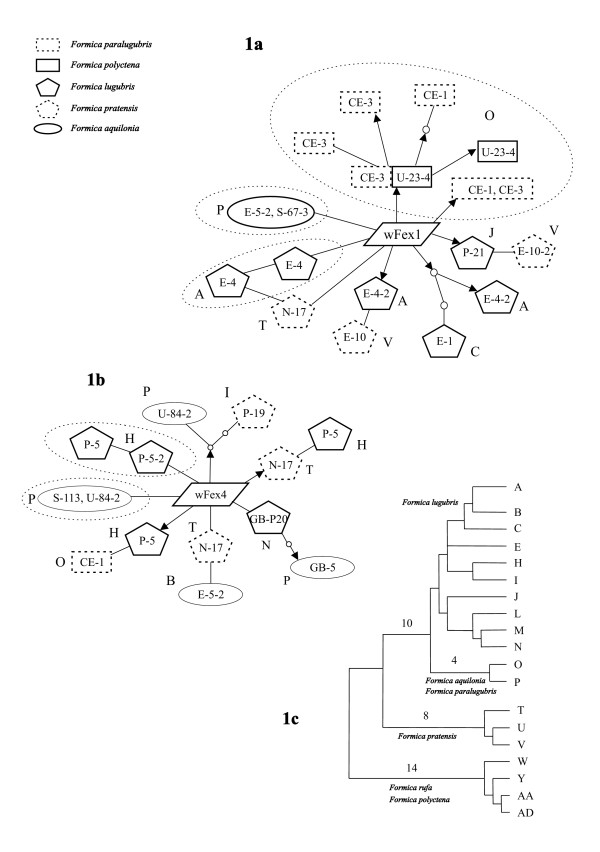
**Haplotype networks of *Wolbachia *strain wFex1 (1a) and wFex4 (1b) *wsp *haplotypes, and the phylogeny of the mtDNA haplotypes (1c) in *Formica rufa *group ants**. Only *wsp *haplotypes that shared mutated nucleotides between two or more ant specimens are included. Lines connecting *wsp *haplotypes represent a single mutation. Arrow lines indicate nonsynonymous substitutions. For each *wsp *haplotype, the box includes the sample code (Table 1) and the letter outside the box gives the mtDNA haplotype. Those parts of the networks that indicate vertical transmission (i.e. related *wsp *haplotypes share the same mtDNA type) are circled with dotted lines. The tree of the mitochondrial haplotypes from [20] includes only haplotypes of the samples used in this study. Numbers above lines indicate how many mutations have taken place in that lineage (numbers above 3 are shown). The figure is not drawn to scale.

The *wsp *haplotype networks showed that shared *Wolbachia *haplotypes were found within as well as between species (Fig. 1a, b). The network of strain wFex1 (Fig. 1a) showed that some haplotypes that shared a common mutation, were associated to an identical mtDNA haplotype (haplotype O from the samples CE-1 and CE-3 of Swiss *F. paralugubris *and U-23-4 of Russian *F. polyctena*, haplotype A from the samples E-4 of *F. lugubris *in Baikal, and haplotype P from the samples E-5-2 and S-67-3 of Russian and Scandinavian *F. aquilonia*). There were also cases in which two *wsp *haplotypes shared a common mutation but were associated to mtDNA haplotypes that were not closely related and not even from the same species (e.g. sample P-21 from French *F. lugubris *and E-10-2 from Siberian *F. pratensis *that were associated to mtDNA haplotypes J and V, respectively). There were only two cases in wFex4 (Fig. 1b) where *wsp *haplotypes that shared a common mutation were associated to a same mtDNA haplotype (haplotypes H and P).

Possible association of the *wsp *haplotypes and the mtDNA haplotype lineages was examined as follows. (1) The *wsp *sequences that differed from the most common type by sharing identical nucleotide variants were considered to form a cluster (putatively a clade). There were eight and seven such clades in wFex1 and wFex4 strains, respectively (Fig. 1a, b). (2) The distances between all pairs of mtDNA haplotypes were calculated as the number of nodes separating the haplotypes in the mitochondrial phylogenetic tree [Fig. 2 in ref. [20]]. (3) For each *wsp *clade we calculated the mean distance between the associated mtDNA haplotypes. (4) The overall congruence of the *wsp *and mtDNA sequence divergence was calculated as the sum of these mean distances over all the clades. (5) The significance of the congruence was tested by permuting the mtDNA haplotypes while keeping the *wsp *network fixed, and recalculating the distances. The significance of the observed association was inferred by repeating the permutations for a thousand times. Using this method, we found a significant association between *Wolbachia *haplotypes and mtDNA haplotypes for the strain wFex1 (P = 0.04) but not for the strain wFex4 (P = 0.30).

### Recombinant sequences

Sequence alignments and comparisons of the *wsp *sequences revealed haplotypes that looked as if recombinants between wFex1 and wFex4 in samples of *F. aquilonia *and *F. polyctena*. Since all were found only once among the initially sequenced clones, 84 additional clones were sequenced from three of samples. Despite the increment, none of the putative recombinant strains were rediscovered. To investigate whether the putative recombinants were true or PCR artifacts we designed PCR primers which amplify wFex1-wFex4 recombinants (forward: 5'-GGTATTGCACATAAATCAGGCA-3' and reverse 5'-AAACTGCATGTCCTCCTTTATC-3') and wFex4-wFex1 recombinants (forward 5'-GGGACTGATGATGTTGATCCT-3' and reverse 5'-TTGAGTTCCCCTTTGCCGTC-3'). The PCR assays did not result in correctly sized bands in any of the original samples when the products were run on an agarose gel, suggesting that the observed wFex1-wFex4 recombinants were PCR artifacts. However, the additional sequencing also identified in *F. aquilonia *a haplotype that was 99.3% similar to strain wFex3 from the ant *F. exsecta *[22]. The strain wFex3 has been characterized as a recombinant between wFex2 and wFex4 [22]. As we did not find the strain wFex2 in any of our *F. aquilonia *samples, the new haplotype cannot be explained as a PCR artifact. Therefore, this haplotype represents an additional infection by a strain which is a variant of the previously described recombinant strain wFex3 or a novel natural recombinant between wFex2 and wFex4.

## Discussion

The major finding of this study is the extensive sharing of *Wolbachia *infections between species of *F. rufa *group wood ants. All the specimens analysed were infected, most of them with at least two different strains, and all the species carried the same two *Wolbachia *strains wFex1 and wFex4, even the same *wsp *haplotypes of the strains. In addition, *F. aquilonia *and *F. rufa *had strains (one and two, respectively) that were not detected in the other species. The widespread infection gives no support to the hypotheses that the infection states might depend on the social structure of the species [16,17]. Our samples included species that have commonly a single queen in a colony (e.g. *F. rufa*, *F. pratensis*) and species that build large networks of colonies with hundreds of queens (*F. aquilonia*, *F. paralugubris*, *F. polyctena*).

Two possible scenarios can explain the sharing of *Wolbachia *infection in the wood ants. First, *Wolbachia *infection could have occurred in a common ancestor predating the speciation of *F. rufa *group ants. This would mean that the two *Wolbachia *infections have been maintained stably in the ants for a long period of time, more than half a million years [20]. Alternatively, *Wolbachia *infections could have been transmitted horizontally between the wood ant species.

Under the hypothesis that the *Wolbachia *infections are ancestral, predating the divergence of the host species, we might expect some degree of evolutionary divergence between the independently evolving *Wolbachia *lineages. However, no fixed nucleotide substitutions were observed in *wsp *sequences from the different ant species. We can use some simple calculations to evaluate whether this lack of divergence is still compatible with independent evolution of the gene lineages. The probability P_0 _of observing no substitutions in either of the two shared strains wFex1 and wFex4 when the expected number is k per strain can be obtained from the Poisson distribution as P_0 _= e^-2k^. Setting P_0 _= 0.05, the standard significance threshold, the limiting value of k is 1.5. This means that we can reject the hypothesis of a single ancestral infection if the expected number of nucleotide substitutions in *wsp *in the internal branches is k ≥ 1.5. The 2051 bp long mtDNA sequences of the *Formica rufa *group had a total of 36 nucleotide substitutions in the internal branches of the haplotype network [20]. This means that for the probability of not observing any taxon specific substitutions in either of the two 600 bp *wsp *sequences to exceed 5%, the *wsp *gene has to evolve at least seven times more slowly than the ant mtDNA [(36/2051)/(1.5/600) = 7].

Wenseleers [13] have estimated that the *wsp *gene in *Wolbachia *infecting ants evolve at a rate of 0.2% per million years (MY), which is roughly ten times more slowly than the rate of 2% per MY estimated for mtDNA in *Drosophila *[23]. Given these estimates, the persistence of ancestral *Wolbachia *infections appears plausible. However, *Wolbachia *with identical *wsp *sequences (both wFex1 and wFex4) have also been found in the ant *F. exsecta *[22], which has been separated from the *F. rufa *group by 3.5 – 4 million years [21]. A calculation similar to that above shows that here the evolutionary rates of host mtDNA and *Wolbachia wsp *have to differ by a factor 22 to be consistent with old ancestral infections.

Even though the above rate estimates include some uncertainty, we can conclude that the sharing of identical *Wolbachia *infections between *F. exsecta *and the species of the *F. rufa *group is more likely to be the result of horizontal transfer than the persistence of an ancestral infection. The horizontal transfers need not be recent as the *wsp *gene likely evolves more slowly than mtDNA and the *Wolbachia *infections may have remained unchanged for some time. However, we cannot exclude the possibility that ants of the *F. rufa *group share an ancestral *Wolbachia *infection with strains wFex1 and wFex4, with neither strain fixing mutations since the split of the host species. Particularly, the phylogenies of wFex1 and mtDNA showed significant, although weak congruence.

When considering only those *wsp *mutations which are shared between different ant samples, we may try to evaluate how frequently horizontal transmission has occurred. Some of the *wsp *haplotypes are shared between samples with the same mitochondrial haplotype. These include sharing of wFex1 haplotypes by *F. aquilonia *samples with a mtDNA haplotype P in Sweden (S-67-3) and Siberia (E-5-2), and by *F. polyctena *from the Urals and *F. paralugubris *from Switzerland carrying the mtDNA haplotype O, and by *F. lugubris *from Siberia with mtDNA haplotype A. The mtDNA haplotypes A and C (in *F. lugubris *from Siberia) also carry related wFex1 haplotypes that could originate from the same ancestor, assuming that this haplotype has been lost from the sample with mtDNA haplotype B (or occurs with such a low frequency that it was not detected by us). Haplotypes of the strain wFex4 show only two cases where ant samples with the same mtDNA haplotype share a *wsp *haplotype, namely *F. aquilonia *from Sweden (S-113) and the Urals (U-84-2) that carry the haplotype P, and *F. lugubris *from France with mtDNA haplotype H. In these cases, vertical transmission is the most likely explanation for the sharing of *wsp *haplotypes.

There are, however, *wsp *haplotypes that are shared between samples that have mitochondrial haplotypes separated by more than 20 mutations (Fig. 1a, b, c). If these cases were explained by vertical transmission of an ancestral *wsp *haplotype, we would have to invoke repeated *wsp *haplotype loss in other mitochondrial lineages that originate from the same ancestor. In addition to this frequent haplotype loss, we would need to assume that the *Wolbachia *infections in the ants have retained ancestral polymorphism, maintaining both these *wsp *haplotypes and the common haplotypes of wFex1 and wFex4 that were found from all samples. These requirements are strong and it is more plausible that sharing of mutated *wsp *haplotypes by different mitochondrial haplotypes has resulted from frequent horizontal transfer of *Wolbachia*. The haplotype networks had eight clear cases of this type, three in wFex1 (including the haplotype pairs J & V, A & V and A & T) and five in wFex4 (including the haplotype pairs I & P, T & H, N & P, T & B, and H & O). The inferred number of horizontal transfers may greatly underestimate the actual number, as is known to be the case for recombination events [24].

Horizontal transmission is not as rare as previously thought [25-27], and has been suggested to have occurred relatively frequently in the fire ants [28]. In the case of vertical transmission, the absence of *wsp *haplotypes from other samples of the same matriline could be due to haplotype loss or failure to amplify low frequency variants. Strain loss has been suggested to have occurred in the fire ants *Solenopsis invicta *and *S. richteri *[17], and in the Argentine ant *Linepithema humile *[19]. In both cases the loss has been connected to invasion of new habitats, and the loss may have taken place either through founder effects or through environmental curing due to the sudden change in climate and/or diet. *F. rufa *group ants have colonized Eurasia before and during the last glaciation [20]. As a consequence there has been ample opportunity for both founder effects and climatic changes to cause the loss of infections. However, the association of *wsp *haplotypes and mtDNA haplotypes point more strongly towards recent horizontal transmission than selective strain loss of an ancestral *wsp *haplotype from some mitochondrial lineages, particularly in wFex4.

As mentioned above, horizontal transmission has evidently happened between different *Formica *ants as well as between ants and other insects. According to blastn searches in GenBank, *Wolbachia *strain wFex1 is identical with strain wDes from the tephritid fruit fly *Dacus destillatoria *[29], and differs by only 0.6% from a strain identified from the raspberry fruit worm *Byturus unicolor *[30]. In addition to wFex1 and wFex4, *F. rufa *contained two other strains, wFex2 and wFrufa. The closest matches of wFex2 in a blastn search were strains from a butterfly *Nymphalis xanthomelas *[31], with a mere 0.4% sequence difference and from a leaf-cutting ant *Acromyrmex insinuator *[32] with a 1.1% difference. The strain wFrufa differed 1.4% from group B *Wolbachia *strain wSdagB1 from the ant *Solenopsis daguerrei *and a strain wDiaspp2 from the spider *Diaea *sp. r1 NSI [33,34]. In contrast to the incidence of wFex1, strain wFex4 has thus far been described only from the ants *F. truncorum *and *F. exsecta *[14,22]. The strain closest to wFex4 is from a parasitoid wasp *Spalangia cameroni *with 6.8% sequence difference.

Some of our samples contained unique *Wolbachia *strains. The presence of wFex2 and wFrufa exclusively in *F. rufa *indicates that these strains have invaded *F. rufa *relatively recently after the closely related mitochondria of *F. rufa *and *F. polyctena *have diverged. Another possibility is that wFex2 and wFrufa have been lost from the other species. Similar arguments hold for the strain variant of wFex3 found in Russian *F. aquilonia*.

All the wFex1-wFex4 recombinant strains detected in our assay turned out to be likely PCR artifacts, even though recombinants between strains have been detected in ants [22]. The fact that the simultaneous amplification of very similar sequences can lead to the amplification of spurious recombinants is not new [e.g. [35]]. Our results indicate that when multiple *Wolbachia *infections are assayed by PCR, special attention should be paid to the presence of possible recombinant strains. In a symbiont which is known to recombine, additional diagnostic tests should be applied in order to separate true from false recombinants.

## Conclusion

We have studied *Wolbachia *transmission dynamics in *Formica *wood ants by comparing *Wolbachia *strains with the associated host mtDNA haplotypes. Our results show that both horizontal and vertical transmission of *Wolbachia *have been important in the infection history of these ants. We found extensive sharing between ant species not only of the same *Wolbachia *strains (wFex1 and wFex4) but even of identical *wsp *haplotypes of these strains. The presence of identical *wsp *haplotypes of the two common *Wolbachia *strains also in a more distant species *F. exsecta *gives support to the hypothesis that horizontal transmission has been an important factor behind the uniform distribution of *Wolbachia *strains in *Formica *ants. In addition, the finding that *Formica *wood ants with distant mtDNA haplotypes share the same rare *wsp *haplotypes is more plausibly explained by horizontal transmission. However, the significant, although weak, congruence between the phylogenetic networks of mtDNA and *wsp *of wFex1 also suggests the significance of vertical transmission. In addition to the two prevailing strains, three separate strains were found in eastern Russia, pointing to the existence of geographically restricted distribution of some strains. The fact that all the ants were infected irrespective of the family structure of their societies gives no support to the proposed hypotheses that the spreading of *Wolbachia *in ants might be associated to the types of their societies.

## Methods

### Samples

The ant species used in this study were *F. aquilonia, F. lugubris, F. paralugubris, F. polyctena*, *F. pratensis *and *F. rufa*, which belong to the *F. rufa *group ants [36]. The 32 worker ant DNA samples and their mtDNA haplotypes were attained from the study of Goropashnaya, Fedorov and Pamilo [20]. The samples, their geographical locations and the mtDNA haplotypes are listed in Table 1.

### *Wolbachia *strain identification

A fragment of the gene encoding the *Wolbachia *surface protein WSP was amplified by PCR from the 32 ant samples using primers wsp81F and wsp691R [37]. Cycling conditions were 95°C for 5 min followed by 45 cycles of 95°C for 30 s, 55°C for 30 s and 72°C for 1 min and finally 72°C for 5 min. A negative control was included in the PCR. Approximately 5 μl of the obtained PCR product was run on a 1.5% agarose gel to ensure the correct size of the amplified fragment. Remaining PCR products were purified with a MinElute PCR Purification Kit (Qiagen, Hilden, Germany) and cloned with a TOPO TA Cloning Kit (Invitrogen, Carlsbad, CA). Bacterial colonies grown overnight were suspended in 50 μl of sterile water and screened with PCR. A new PCR for sequencing was performed for the positive clones. The PCR product was sequenced on one or both strands using a BigDye Terminator v3.0 Cycle Sequencing Kit (Applied Biosystems, Foster City, CA) and run on an ABI 377 automated sequencer.

Sequences were aligned and compared with the program Sequencher v. 4.0.5 (Gene Codes Corporation). *Wolbachia *strains were identified using blastn searches in GenBank. Haplotype networks were constructed using statistical parsimony method implemented in the program TCS [38].

### Comparison of Wolbachia and mtDNA phylogenies

The possible congruence of the *Wolbachia *and mtDNA phylogenies was examined by testing whether the *wsp *haplotypes that share a variant nucleotide are associated to phylogenetically similar mtDNA haplotypes. This was done by permuting the mtDNA haplotypes (see Results for a detailed description).

## Authors' contributions

LV and MR carried out the PCR assays, cloning and sequencing work. LV did the strain identifications, *wsp *haplotype networks and drafted the manuscript. PP coordinated the study, carried out the comparison of *Wolbachia *and mtDNA phylogenies and helped to draft the manuscript. The final version of the manuscript was read and approved by all authors.
